# Computed tomography-derived bone and muscle biomarkers after fragility hip fracture: readiness for precision risk stratification

**DOI:** 10.3389/fmed.2026.2003981

**Published:** 2026-10-30

**Authors:** Yingsong Yuan, Yunqiang Zhuang, Yang Gu

**Affiliations:** 1 Department of Radiology, Ningbo No.6 Hospital, Ningbo, Zhejiang, China; 2 Ningbo Clinical Research Center for Orthopedics, Sports Medicine & Rehabilitation, Ningbo, Zhejiang, China; 3 Trauma Orthopedics Center, Ningbo No.6 Hospital, Ningbo, Zhejiang, China

**Keywords:** bone mineral density, computed tomography, fragility hip fracture, imaging biomarkers, muscle attenuation, myosteatosis, precision medicine, quantitative imaging

## Abstract

Fragility hip fracture is heterogeneous in local skeletal competence, physiological reserve, and recovery potential. Computed tomography (CT), when already acquired for clinical care, can provide proximal-femoral attenuation or quantitative CT-derived bone mineral density, muscle cross-sectional area, and composition metrics. This Mini Review separates acute fracture-status discrimination, pre-fracture longitudinal prediction, and post-index-fracture prognostication. Local femoral attenuation has been associated with fixation-related complications, but it is an indirect structural marker whose interpretation depends on geometry, loading, reduction, and implant–bone interactions. Muscle composition can complement size, but neither directly measures force, coordination, or functional performance. High apparent discrimination frequently derives from the development sample; evidence of added value over clinical predictors, calibration, and independent validation remains limited. We organize interpretation by anatomy, metric, outcome, and time, and illustrate how structural imaging could be combined with clinical, functional, and biomechanical measurements for four post-fracture endpoints. This is an endpoint-specific evidence interpretation framework, not a validated prediction model or treatment algorithm. Prognostic enrichment must be distinguished from treatment-effect modification. Harmonized measurements, external validation, and prospective evaluation of biomarker-guided decisions are required before these phenotypes can support individualized interventions.

## 1 Introduction

Fragility hip fracture is not a single biological phenotype. Patients with similar fracture morphology may differ substantially in skeletal fragility, local fixation competence, muscle reserve, comorbidity, cognition, and capacity for postoperative mobilization. These differences matter because hip fracture is followed by excess mortality, incomplete recovery to pre-fracture function, and persistent vulnerability to subsequent fracture (1–3). Bone mineral density remains central to osteoporosis assessment, while contemporary sarcopenia frameworks emphasize that muscle strength and performance cannot be inferred from muscle mass alone (4, 5). Rehabilitation after hip fracture is therefore necessarily multidimensional (6).

Computed tomography (CT) creates an opportunity for precision phenotyping because clinically indicated scans may contain quantitative information on bone and soft tissue without requiring an additional acquisition. Opportunistic imaging has expanded from manual measurements to automated extraction of bone, muscle, and adipose-tissue biomarkers (7, 8). At the same time, broad reviews already cover musculoskeletal opportunistic CT, osteoporosis screening, sarcopenia imaging, and secondary fracture risk (9–14). A more focused question is therefore needed: how should CT-derived bone and muscle measurements be interpreted in an older adult who has already sustained a fragility hip fracture?

We use the working term CT-derived bone-muscle phenotype for a quantitative profile combining skeletal and muscular characteristics. Our contribution is an endpoint-specific evidence interpretation framework organized by anatomy, metric, outcome, and time (Figure 1). Figure 1 is a conceptual illustration; its arrows describe a proposed integration of information, not an implemented decision pathway. These dimensions specify which measurement could address which clinical question; they do not constitute a new validated score, establish superiority over existing risk approaches, or demonstrate improved patient outcomes.

**Figure 1:**
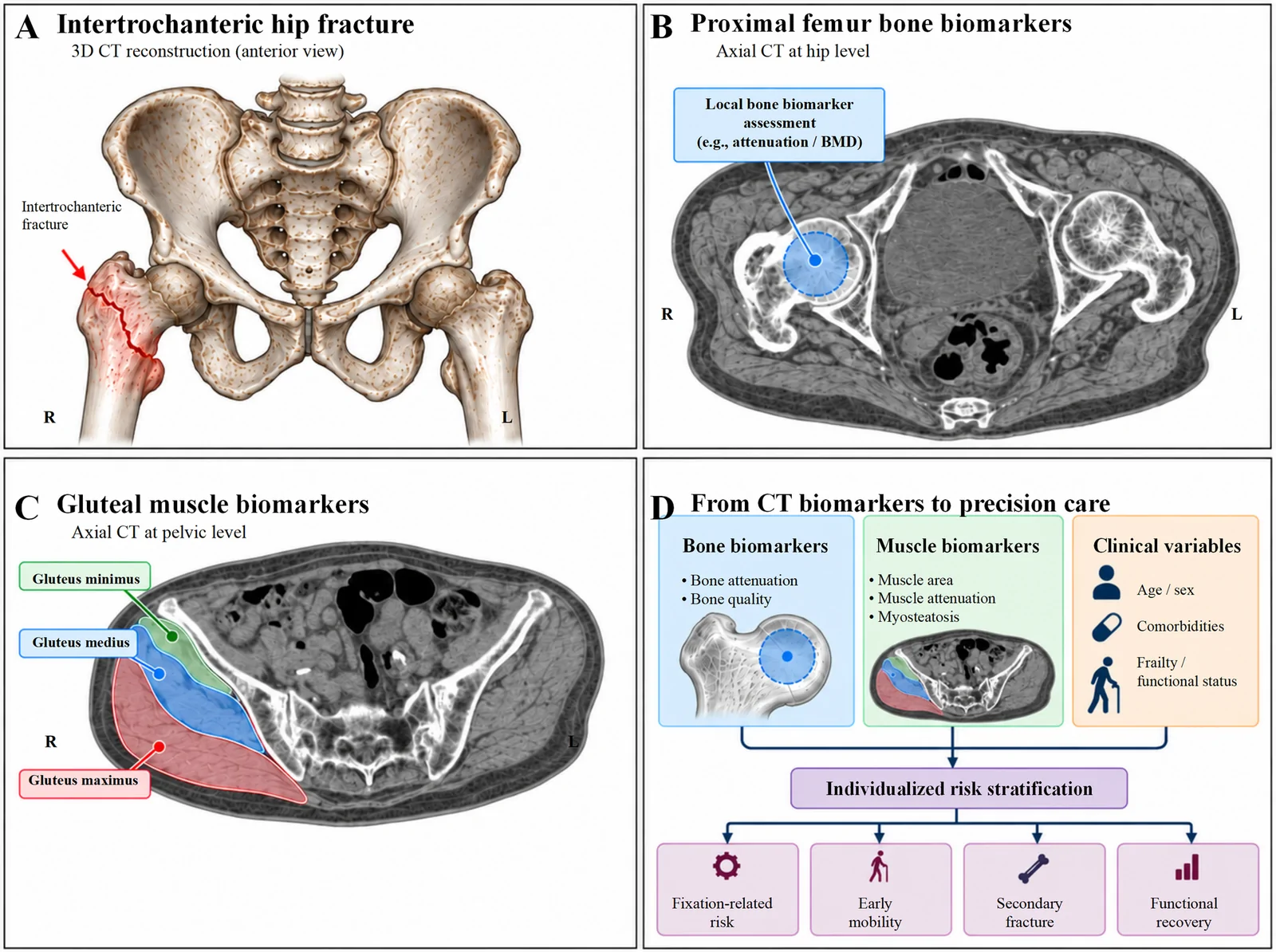
Conceptual integration of CT-derived bone and muscle biomarkers after fragility hip fracture. **(A)** Schematic intertrochanteric fracture. **(B)** Illustrative proximal-femoral bone region of interest. **(C)** Approximate gluteal compartments for muscle assessment. Anatomical and CT-style components are AI-assisted re-creations using real CT images as anatomical references; they are not unaltered diagnostic scans or validated segmentations. Panel C illustrates the relative arrangement of gluteus minimus, medius, and maximus, not exact fascial boundaries. No measurements are derived from the schematic regions. **(D)** Proposed integration with clinical information for four candidate outcome domains. ‘Bone quality’ denotes candidate imaging attributes, not directly measured whole-bone strength. Functional status requires separate clinical or performance assessment; muscle area and attenuation cannot replace strength, balance, or gait testing. Fracture geometry, reduction, implant position, and loading also require separate biomechanical assessment. Arrows and ‘individualized risk stratification’ indicate research aims, not a validated algorithm, established implementation, causality, or treatment benefit. The panels do not constitute a patient case series. Use is limited to clinically indicated CT, without additional acquisition or scan extension solely for phenotyping. BMD, bone mineral density; CT, computed tomography.

## 2 Literature identification and precision-care evidence framework

### 2.1 Literature search and study selection

This purposive Mini Review used a focused narrative search of PubMed/MEDLINE, publisher platforms, and reference lists, with no prespecified lower date limit. The submitted evidence base was updated through 1 September 2026. Principal concepts combined hip fracture, CT/QCT or Hounsfield units (HU), bone or muscle measurements, and fixation failure, mortality, mobility, falls, or second fracture. Supplementary Methods S1 provides reproducible concept strings and distinguishes this search description from targeted primary-source and full-text verification for this revision on 18 October 2026.

Selection was representative rather than exhaustive. We prioritized original human studies with interpretable anatomical sites, measurement definitions, CT timing, and clinically relevant endpoints in hip-fracture patients or older-adult cohorts relevant to the clinical question. Technical and consensus papers were retained when they clarified standardization, automation, or translation. These priorities favor clinically interpretable examples, but do not remove selection bias or establish that the included studies represent all available evidence. Studies lacking these features were not emphasized. Related cohorts were not treated as independent replication. Positive and null findings were considered together, with discrepancies interpreted by site, endpoint, adjustment, and timing rather than significance counting. No dual independent screening, PRISMA flow, formal risk-of-bias scoring, or meta-analysis was performed. Table 1 and Supplementary Table S1 separate unadjusted and multivariable associations from apparent, internally validated, and externally validated prediction.

**Table 1:** Representative CT biomarker studies: outcomes, adjustment, performance, and validation.

Study/design	CT measurement/timing	Endpoint/horizon	Principal finding	Adjustment/key confounders	Performance and external validation
Eto 2026 (20) Measurement study; *n* = 52	Femoral-head HU; neck-axis reformats; preoperative	DXA <60% young adult mean (T-score <−3.3)	Axial AUC 0.832; cutoff 151.5 HU; ICC >0.9	DXA reference; orientation and ROI choice	Sensitivity 0.756; specificity 0.783. Same-sample threshold; no outcome-model validation.
Lund 2020 (21) Retrospective; *n* = 114	Head/neck HU; CT allowed within 6 months before or after surgery	Penetration, shortening, revision	Regional HU associations	Fracture/construct mechanics; mixed CT timing	Association study. Prognostic calibration and independent external validation not established here.
Fan 2023/Ye 2026 (22, 23) *n* = 160/143	Femoral-head/proximal-femoral HU; operative episode	Implant/fixation failure	Apparent AUC 0.724/0.860 (CI 0.778–0.941)	Fan: HU, AO class, reduction. Ye: HU, wall integrity, blood loss, age, sex, side.	No independent external validation of either outcome model; clinical-model increment not reported.
Bae 2021 (25) Retrospective; *n* = 462	L4 psoas attenuation/area index; admission CT	In-hospital mortality; 19 deaths	Attenuation associated; area index univariable null	Combined ROC: age, sex, diastolic pressure, albumin	AUC 0.929 (CI 0.902–0.951), apparent. Clinical-only comparison, calibration and independent validation NR.
Achebe 2026 (29) *n* = 140; final model *n* = 106	Quadriceps HU; preoperative; variable available thigh level	210 observations; day 1/discharge log(feet + 1)	*β*=0.042 per HU (CI 0.012–0.072)	BMI, comorbidity, wheelchair use, timepoint, surgery timing/method	Marginal R^2^ 0.272, not *Δ*R^2^. Intra-reader ICC 0.925; independent outcome validation NR.
Ge 2026 (30) *n* = 590; full model *n* = 484	Gluteal attenuation/area; CT within 72 h of injury	MFES <8/recalled past-year falls	Fear-of-falling OR 0.77 (CI 0.60–0.98) per sex-specific SD	Age, sex, BMI, comorbidity, diabetes, albumin, vision, activity, BMD	Area null after adjustment; past-fall association null after BMD adjustment. No prospective fall prediction.
Mühlberg 2019 (31) 40 fractures/55 controls	Femoral bone plus thigh soft tissue; acute CT	Existing fracture status	Combined tissue information	Age, body composition and acute injury may influence discrimination	Within-sample fracture discrimination; not prospective recurrent-risk validation.
Li 2026 (33) *n* = 550; matched *n* = 320	Phantom-less QCT and gluteal IMAT; index CT	Existing fracture status	Combined AUC 0.927 (CI 0.899–0.954), selected model 2	Age, sex, BMI matching; FN vBMD plus two IMAT measures	Hosmer–Lemeshow *P* = 0.83 in matched data; no independent validation. Not prospective risk calibration.
Pickhardt 2020/Liu 2024 (35, 36) *n* = 9,223/6,926	Abdominal CT; before outcome fractures	Future fracture; 2/10 years	Hip AUC: CT 0.76, FRAX 0.73, CT + FRAX 0.78; Liu 0.733	FRAX/age-sex-matched controls; imaging combinations	No established significant FRAX gain; Liu bone-only AUC 0.702. No post-index validation.
Wang 2022/2024 (37, 38) Related prospective cohort	Contralateral hip BMD/gluteal HU; index CT	Second fracture; median follow-up 4.5 years	45 second fractures; time-dependent associations	Age/sex and mobility/metabolic covariates; later mobility risks information leakage	Cause-specific and Fine–Gray analyses in 2022. Shared cohort is not external validation.

AUC, area under the receiver operating characteristic curve; BMD, bone mineral density; BMI, body mass index; CI, confidence interval; CT, computed tomography; DXA, dual-energy x-ray absorptiometry; HU, Hounsfield units; ICC, intraclass correlation coefficient; IMAT, intermuscular adipose tissue; MFES, modified falls efficacy scale; OR, odds ratio; ROC, receiver operating characteristic; ROI, region of interest; SD, standard deviation; FN, femoral neck; vBMD, volumetric BMD. Apparent performance is evaluated in the development sample. Study-defined thresholds are not treatment rules. Supplementary Table S1 reports effect scales, model samples, clinical adjustment, calibration, and validation in detail. NR, not reported in the inspected primary full text.

### 2.2 What makes a CT biomarker ready for precision care?

Precision-care readiness requires analytical reliability, endpoint-specific association, added predictive value beyond clinical assessment, external calibration, and demonstrated decision benefit. These stages answer different questions: whether the measurement is reproducible at a defined landmark; whether it relates to the intended endpoint and horizon; whether it improves a credible clinical model; whether predictions remain accurate in a new population; and whether using them changes a decision in a way that improves patient-centered outcomes. A technically reproducible value is therefore the starting point, rather than sufficient evidence for individualized care. Unadjusted association establishes neither independence nor prediction; multivariable adjustment addresses measured confounders but is not validation. Apparent performance is estimated in the development sample. Internal validation requires resampling or a held-out sample; external validation requires an independent population. A significant adjusted coefficient, measurement ICC, or validated segmentation algorithm cannot substitute for outcome-model validation.

Clinical-only comparators should include age, fracture morphology, cognition, comorbidity, and pre-fracture mobility as appropriate. Imaging-enhanced models should report changes in discrimination with uncertainty, calibration intercept and slope or plots, and decision-relevant net benefit. Calibration matters particularly for absolute risk: useful ranking can coexist with systematic overestimation or underestimation in another setting. Model evaluation should also specify when each predictor becomes available, so that a preoperative claim does not use postoperative information. Prognostic enrichment identifies higher-risk patients; decision support links an estimate to a specified action; treatment-effect modification requires evidence that treatment benefit differs by biomarker. These are separate claims.

HU, areal BMD, volumetric BMD, cortical thickness, and structural measures quantify different properties (15, 16). Attenuation or calibrated mineral density may inform tissue-level material-property estimates, but neither directly measures stiffness, toughness, or failure load. Whole-bone competence also depends on geometry, cortical distribution, trabecular architecture, and load direction. Patient-specific finite-element models combine geometry and density-to-material assumptions with boundary conditions; estimated strength changes with loading assumptions (17). For fixation, reduction, implant position, interface purchase, and construct loading add further determinants. Simple HU is therefore an indirect indicator; incorporating it into a biomechanical model requires separate model validation.

Muscle area or volume describes quantity; mean attenuation and fat-related metrics describe composition (18, 19). We use muscle attenuation for HU; source-study ‘muscle density’ denotes radiodensity, not physical density. Muscle quality is a broader functional construct. Myosteatosis requires its operational definition, such as low attenuation or a segmented fat fraction. CT timing and endpoint horizon must be specified: pre-fracture CT, index-fracture CT, and postoperative CT address different questions. For example, a preoperative scan may inform a later outcome, but a postoperative measurement cannot retrospectively support a preoperative choice. Early walking distance, discharge independence, later functional recovery, and second fracture are not interchangeable endpoints, even when the same muscle or bone region is measured.

## 3 Evidence organized by clinical question and time

Three evidence streams are distinguished: fracture-status discrimination, pre-fracture longitudinal prediction, and post-index prognostication. The last includes mechanically and functionally distinct outcomes. Their associations and discrimination statistics do not establish treatment thresholds.

### 3.1 Post-index prognostication: local skeletal competence and fixation-related risk

Post-fracture anatomy makes local skeletal measurement clinically attractive but technically difficult. In 52 intertrochanteric fractures, Eto et al. aligned reconstructions to the femoral-neck axis and reported measurement ICCs above 0.9. Their axial cutoff of 151.5 HU identified study-defined severe osteoporosis (DXA <60% of young adult mean; T-score <−3.3), with AUC 0.832, sensitivity 0.756, and specificity 0.783 (20). This validates neither implant selection nor augmentation benefit. Its principal contribution is a reproducible measurement approach tied to a study-specific DXA category, rather than a universal mechanical-failure cutoff.

Displacement, rotation, and comminution can cause nominally identical slices to sample different tissue. Protocols should specify an intact landmark, fractured or contralateral side, neck-axis reformation, and exclusion of cortex or fracture gaps from trabecular ROIs. Contralateral measurements describe the opposite side and cannot automatically represent local implant purchase.

In 114 femoral-neck fractures, Lund et al. associated regional HU with screw penetration or shortening, although CT was permitted before or after surgery (21). Fan et al. reported an apparent implant-failure AUC of 0.724 in 160 patients; Ye et al. reported 0.860 (95% CI 0.778–0.941) in 143 patients (22, 23). Different regions, failure definitions, and selected samples preclude direct ranking. Both reported adjusted HU associations, but neither demonstrated incremental performance over a validated mechanical/clinical model or independent external validation.

Comparable HU can coexist with different failure risks in stable, well-reduced constructs and unstable, poorly reduced fractures. The next step is an outcome-specific model that tests incremental value over mechanical assessment, including fracture morphology, reduction quality, and implant position. A local density measurement cannot summarize the distribution of load throughout the bone–implant construct. Current evidence cannot justify choosing fixation, arthroplasty, implant design, or cement augmentation from HU alone.

### 3.2 Post-index prognostication: muscle biomarkers, physiological reserve, and functional recovery

Muscle findings vary by site and horizon. Boutin et al. linked trunk-muscle measurements to survival (24). Bae and Lee found an adjusted association between psoas attenuation and in-hospital mortality, whereas area index was not associated in univariable analysis; their combined AUC of 0.929 arose from only 19 deaths and lacked a clinical-only comparator (25). A prospective study associated gluteal attenuation with earlier mortality and gluteus maximus area with later mortality (26). These markers may reflect systemic vulnerability as well as local function. Psoas and paravertebral measures should therefore not be assumed to carry the same functional meaning as gluteal or quadriceps measures. The differing area and attenuation findings argue against a universal best muscle or a single static definition of muscle quality.

Suto et al. associated selected muscle areas with ambulatory status (27), whereas Yokoyama et al. found gluteal attenuation, but no examined area measure, associated with six-month walking category (28). These clinical classifications differ from instrumented gait testing. Achebe et al. recorded physical-therapy walking distance on postoperative day 1 and at discharge. Their final quadriceps model included 106 patients and 210 observations; *β*=0.042 (95% CI 0.012–0.072) per HU refers to log(feet+1), not feet walked (29). Ge et al. assessed fear of falling and recalled past-year falls. In 484 complete cases, gluteus medius/minimus attenuation was associated with fear of falling (OR 0.77, 95% CI 0.60–0.98 per sex-specific SD increase), but the BMD-adjusted past-fall association was not significant; area measures were also null after adjustment (30). Neither endpoint demonstrates prospective fall prediction.

Morphology does not directly quantify force-generating capacity, motor recruitment, coordination, dynamic joint stability, or activities of daily living. Similar CT profiles can accompany different pain, cognition, balance, and movement strategies (5, 18, 19). Research should pair imaging with pre-fracture mobility history, feasible dynamometry, balance testing, gait speed, and standardized performance measures. Instrumented gait or wearable measures can characterize symmetry and joint kinematics; force measurements and electromyography can assess kinetics and activation when feasible. Assessment timing and weight-bearing restrictions matter. When acute pain or postoperative restrictions prevent a test, that inability should be recorded rather than interpreted as a normal result; later reassessment may answer a different recovery question. Poor-outcome prognosis does not show who benefits from greater rehabilitation intensity, which requires comparative intervention evidence.

### 3.3 Fracture-status discrimination at the index event

Mühlberg et al. combined femoral bone and thigh soft-tissue descriptors to distinguish existing fractures from controls (31). In Wang et al., gluteus medius/minimus attenuation yielded AUCs of 0.88 (95% CI 0.85–0.92) for femoral-neck fracture and 0.95 (0.92–0.97) for intertrochanteric fracture; combined bone-muscle models yielded 0.912 and 0.958 (32). Li et al. analyzed 320 participants after matching. Femoral-neck vBMD plus two gluteal fat measures yielded AUC 0.927 (95% CI 0.899–0.954), with Hosmer–Lemeshow *P* = 0.83 (33). This same-sample goodness-of-fit test does not establish transportable absolute-risk calibration. Automated pelvic-muscle analyses offer further phenotyping (34), but these are acute fracture-control comparisons.

Imaging acquired at the fracture cannot prospectively predict that event. Same-sample AUC gains over bone-only models are not equivalent to added value over a clinical risk model, calibrated future-fracture probabilities, or improved treatment outcomes. Case-control sampling and acute injury further constrain transportability.

### 3.4 Pre-fracture longitudinal prediction

Pre-fracture CT provides temporal evidence, although these cohorts need not exclude all previous fractures. In Pickhardt et al., two-year hip-fracture AUC was 0.76 (95% CI 0.67–0.86) for bone/muscle attenuation versus 0.73 (0.60–0.86) for FRAX (*P* = 0.73). The CT-plus-FRAX model reached 0.78 (0.68–0.88), without demonstrated significant improvement (35). In Liu et al., ten-year AUC increased from 0.702 for bone attenuation to 0.733 for bone, muscle, and subcutaneous fat (36). The latter comparison concerns imaging combinations, not improvement beyond a complete clinical model. Neither establishes transportability to post-index hip-fracture care; an externally tested segmentation algorithm does not validate a prognostic equation.

### 3.5 Post-index prognostication: second hip fracture and time-dependent risk

Wang et al. associated lower gluteus medius/minimus attenuation with second fracture over median 4.5-year follow-up (HR 1.68, 95% CI 1.20–2.35 per SD decrease); femoral-neck aBMD was not significant across that horizon (37). Their related time-window analysis suggested earlier relevance of femoral-neck aBMD and later relevance of muscle attenuation (38). Only 10 second fracture occurred in year 1, limiting precision. Together with the meta-analysis (14), these findings support time-aware hypotheses, not independent replication or treatment-response prediction.

Death prevents subsequent fracture. Wang et al. included cause-specific models and Fine–Gray sensitivity analyses (37). Cause-specific hazards describe rates among patients still event-free; cumulative-incidence estimates, including Fine–Gray modelling, address absolute fracture probability with competing death (39). Future models should prespecify the estimand and horizon, validate cumulative-incidence calibration, and avoid interpreting Kaplan–Meier complements as observed fracture probability. Predictors measured after baseline, including later mobility, must not be used as if available for an index-fracture decision.

## 4 Discussion: from association to individualized care

Operational use begins with a prespecified endpoint and horizon, followed by a feasible measurement and a clinical-only comparator. For fixation failure over 6–12 months, local femoral HU/vBMD and cortical distribution would supplement fracture stability, reduction, implant position, and loading. For early mobility from postoperative day 1 to discharge, available quadriceps attenuation would supplement pre-fracture mobility, pain, cognition, and permitted weight-bearing, with observed walking distance as the endpoint. For function at 6–12 months, gluteal quantity/composition would accompany serial strength, balance, gait, and patient-reported daily function. For second fracture at 1, 2, or 5 years, contralateral hip bone and gluteal measures would supplement prior fractures, falls, mobility, treatment, and competing mortality. These are proposed research specifications, not validated assignments of exclusive anatomical roles.

For example, consider a hypothetical previously ambulatory older adult with an intertrochanteric fracture and clinically indicated preoperative CT showing low local bone attenuation and, if the available scan covers the prespecified quadriceps level, low quadriceps attenuation. A research workflow would first document scan validity, fracture stability, cognition, pain, and baseline mobility. It would then compare clinical-only and imaging-enhanced predictions of discharge walking distance, using postoperative reduction and weight-bearing information only at an explicitly postoperative decision point. A future trial could test whether disclosure improves rehabilitation assessment or referral. The candidate action would be a structured multidisciplinary assessment of modifiable barriers to walking, with usual clinical assessment as the comparator. Whether imaging changes referral decisions, improves discharge function, or merely duplicates available information would be measured prospectively. The example assigns no risk probability, implant choice, cement indication, or rehabilitation dose.

Technical effects differ by tissue (40–43). Contrast and tube voltage alter HU in both bone and muscle; enhancement can obscure low muscle attenuation, and contrast corrections require protocol-specific validation (44). Reconstruction kernels and noise affect attenuation distributions and segmentation. Partial volume particularly compromises thin cortices and muscle–fat boundaries; slice thickness changes apparent cortical thickness and fat fractions. Bone protocols must distinguish trabecular ROIs from cortex and calibrated vBMD from raw HU. Muscle protocols must specify fascia, visible fat inclusion, thresholds, side, and exclusion of edema or hematoma. Minimum reporting should include scanner, kVp, contrast phase, kernel, voxel size, anatomical landmark and side, software/version, calibration, normalization, time from injury or surgery to CT, missing coverage, and segmentation failures. The eligible, successfully measured, and final analytical sample sizes should be distinguishable. Cross-scanner harmonization should examine agreement of absolute values, not merely correlation. Repeatability under unchanged conditions, interobserver agreement on the same images, and interscanner agreement under differing hardware/protocols are separate properties; a high reader ICC establishes neither scan–rescan repeatability nor cross-scanner interchangeability.

Consensus supports imaging alongside functional assessment (45). Automation and phantom-less QCT improve feasibility but do not remove these requirements (46, 47). Clinical deployment additionally requires image verification, referral ownership, and workflow integration (48). Segmentation success should be reported for all eligible scans, because excluding failed or incomplete examinations may select a technically easier or clinically different subgroup. CT should supplement established surgical, geriatric, nutritional, and rehabilitation assessment. Secondary fracture prevention is already indicated by the index event and should not await an imaging threshold (49). Extract biomarkers only from clinically indicated CT; do not acquire or extend CT solely for phenotyping.

After external validation against strong clinical and functional comparators, prospective impact studies should test a specified biomarker-guided decision against usual care, preferably with randomized allocation when feasible. Outcomes should include function, complications, quality of life, harms, and workload. Potential harms include false reassurance from apparently favorable imaging, unnecessary escalation after a low attenuation value, and unequal access to measurement or follow-up. A useful strategy must improve care sufficiently to justify these burdens. Treatment-effect modification requires prespecified biomarker-by-treatment interaction analyses in comparative intervention data. A biomarker-guided strategy trial instead tests whether using the marker improves care. Poor prognosis alone cannot identify benefit from intensive rehabilitation or altered surgery. Serial functional assessment may improve triage even when CT adds little. This possibility and null incremental findings must remain explicit.

## 5 Conclusion

Clinically indicated CT contains skeletal and muscle information beyond fracture morphology. The evidence supports candidate structural and compositional biomarkers, with substantial variation by anatomy, endpoint, and time. Fracture-status discrimination, pre-fracture longitudinal prediction, and post-index prognosis remain distinct.

The proposed framework organizes evidence and future investigation. It does not establish a validated treatment-selection method. Progress requires reproducible measurements, integration with biomechanics and objective function, incremental prediction and calibration, independent validation, and prospective evaluation of decision benefit. Until those steps are completed, quantitative CT should complement established surgical assessment, geriatric evaluation, rehabilitation planning, and secondary fracture prevention. Its value remains opportunistic: no additional CT acquisition or scan extension should be performed solely for biomarker generation.
