# Supplementary material for: Computed tomography-derived bone and muscle biomarkers after fragility hip fracture: readiness for precision risk stratification
Source: Front Med (Lausanne). 2026 Oct 30;13:2003981. doi: 10.3389/fmed.2026.2003981 (PMC13760348; doi:10.3389/fmed.2026.2003981)
Supplement: Supplementary file 1 [file Supplementaryfile1.docx]

Supplementary material

Manuscript 2003981 | Supplementary Methods S1 and Table S1

## S1 Scope and search approach

This purposive narrative review separates acute fracture-status discrimination, pre-fracture longitudinal prediction, and post-index-fracture prognosis. The evidence base in the submitted manuscript was updated through 1 September 2026. The reproducible concept strings below reconstruct the reported search scope; they are not a recovered verbatim historical search log. Targeted verification of cited primary records and full texts was completed on 18 October 2026. This was not a new exhaustive database search and does not imply complete coverage through October.

## S1.1 Principal concept strings

The following strings can be entered in PubMed without a lower publication-date limit. They describe the principal clinical/measurement concepts; terminology and reference-list searching supplement them.

## Query 1

("hip fracture" OR "femoral neck fracture" OR "intertrochanteric fracture") AND ("computed tomography" OR "quantitative CT" OR "Hounsfield units") AND ("bone mineral density" OR cortical OR "muscle attenuation" OR "muscle density" OR myosteatosis)

## Query 2

("hip fracture" OR "femoral neck fracture" OR "intertrochanteric fracture") AND ("computed tomography" OR "Hounsfield units") AND ("fixation failure" OR mortality OR ambulation OR walking OR "second hip fracture")

## Targeted technical concepts

Finite-element models and proximal-femoral loading; CT contrast and tube-voltage effects; muscle segmentation and anatomical landmarks; competing-risk models and second hip fracture. Methodological references supplement the clinical evidence.

## S1.2 Selection and interpretation

Original human studies were prioritized when CT anatomy, metric, acquisition timing, and endpoint were interpretable. Acute fracture-control studies, pre-fracture longitudinal cohorts, and post-index cohorts were considered separately. The pre-fracture stream is not restricted to people without any previous fracture. Technical and consensus studies informed measurement interpretation. Selection was representative, without dual independent screening or formal risk-of-bias scoring; no screening denominator or PRISMA flow is claimed. Null findings, limited adjustment, incomplete imaging coverage, and discordant endpoints were retained. Related CASH/CSHFE reports were not treated as independent replication.

## S1.3 Extraction and evidence levels

Table S1 records analytical sample sizes, CT timing, endpoints, adjustment, effect scales, discrimination, comparator models, calibration, and validation. Quantitative details were checked against primary full texts; analytical samples were distinguished from full cohorts. An adjusted association is distinct from a prediction model. Apparent performance uses development data; internal validation requires resampling or a held-out sample; external validation tests the outcome model in an independent population. Image-processing validation and reader reliability are separate. Confidence intervals or calibration measures were not imputed.

## S1.4 Reporting limitations

NR means not reported in the inspected primary full text; it does not establish absence from every related supplement. Calibration is described by the measure actually reported. A nonsignificant Hosmer–Lemeshow test in a matched case-control sample does not demonstrate accurate absolute risk in a target population. R² is not ΔR², and an imaging-only comparison does not establish incremental value over clinical prediction. No meta-analysis or cross-study AUC ranking was performed. References follow main-manuscript numbering.

# Table S1 Quantitative evidence and validation status

95% confidence intervals are shown where available. Definitions and reporting limitations are stated in Methods S1. Values are not directly comparable across different endpoints and horizons.

| **Study / target** | **Design and CT timing** | **Adjustment / analytical level** | **Effect or discrimination** | **Increment / calibration** | **Validation and interpretive limit** |
| --- | --- | --- | --- | --- | --- |
| Eto 2026 (20) | n=52; 29 severe / 23 non-severe; preoperative head HU, neck-axis reformats; contralateral DXA <60% young adult mean (T-score <−3.3). | 120 kV; 1-mm reconstructions. Two readers repeated measurements after 3 weeks. Correlation, ICC, Bland–Altman and ROC. | Axial AUC 0.832 (CI NR); cutoff 151.5 HU: sensitivity 0.756, specificity 0.783. Fracture-side axial inter-reader ICC 0.987 / 0.994. | No clinical-outcome increment tested. Same-sample threshold; prognostic calibration not evaluated. | Reader agreement, not scan–rescan or interscanner validation. No independent validation or evidence of augmentation benefit. |
| Fan 2023 (22) | Retrospective, n=160; 15 implant failures; perioperative head HU | Multivariable logistic model retained HU, AO class and reduction quality. | HU OR 0.972 (CI 0.952–0.993) per HU; poor reduction OR 7.614 (1.390–41.717). HU AUC 0.724; AUC CI NR. | ΔAUC over clinical-only model NR; calibration NR. Cutoff 124.85 HU was selected in the same data. | Apparent ROC, no resampling correction or independent validation reported. Few events; surgical factors remain material. |
| Ye 2026 (23) | Retrospective, n=143; 21 failures; median follow-up 26 months; proximal-femoral trabecular ROI | Logistic model included HU, posteromedial wall integrity, blood loss, age, sex and fracture side. | OR 0.949 (CI 0.920–0.979) per HU; apparent AUC 0.860 (0.778–0.941). | Clinical-only comparison and calibration NR. Selected 27.6 HU cutoff: sensitivity 81%, specificity 86%. | No independent external validation. Different ROIs and selected samples preclude using these cutoffs interchangeably. |
| Bae 2021 (25) | Retrospective n=462; 19 in-hospital deaths; bilateral L4 psoas HU and area/height² on admission CT. | Univariable screening → logistic model: age, sex, CCI, DBP, BUN, creatinine, albumin, troponin-T, hospital days, HU. Combined ROC: HU + age, sex, DBP, albumin. | HU OR 0.82 (CI 0.74–0.90) per HU. AUC: HU 0.839 (0.803–0.872); combined 0.929 (0.902–0.951). Area index: univariable P=0.890. | Clinical-only AUC and calibration NR. Marker-only versus combined AUC does not isolate CT added to clinical prediction. | Apparent performance; resampling and independent validation NR. Only 19 deaths. Hospital duration in the association model is unavailable at admission. Inter-reader HU ICC 0.908–0.938. |
| Yokoyama 2025 (28) | Retrospective n=58; 41 walking / 17 non-walking at 6 months; unaffected-side CT before hemiarthroplasty | Group comparisons and single-marker ROC; no multivariable clinical prediction model. | Gluteus medius HU AUC 0.86; maximus 0.77. Area AUCs 0.55–0.58 and not significant. AUC CIs NR. | Increment over clinical predictors and calibration NR. | Apparent cutoff optimization; no internal correction or external validation reported. Walking category is not instrumented gait; strength/balance not directly tested. |
| Achebe 2026 (29) | Retrospective n=140; quadriceps HU available in 112; final model 106 patients / 210 observations. PT-recorded walking distance at day 1 and discharge. | Random-intercept model of log(feet + 1): quadriceps HU, BMI, CCI, preinjury wheelchair use, timepoint, time to surgery, fixation method. | β=0.042 (CI 0.012–0.072) per HU on log(feet + 1) scale. Marginal R²=0.272; conditional R²=0.762. Other whole-cohort muscle metrics nonsignificant. | No clinical-only ΔR², out-of-sample error or calibration reported. Conditional R² includes patient random effects; it is not preoperative predictive performance. | Intra-reader ICC 0.925 (0.823–0.969); cross-level HU CV 16.5–17.7%. Inter-reader and independent outcome validation NR. Variable quadriceps levels and incomplete coverage limit transferability. |
| Ge 2026 (30) | Cross-sectional n=590; full model n=484 owing to 106 missing BMD values. CT ≤72 h; unaffected-side gluteal/thigh measurements. FOF: MFES <8; recalled ≥1 fall in prior year. | Sex-specific Z scores. Model 1: age, sex, BMI; model 2 adds CCI, diabetes, albumin, visual impairment, PASE; model 3 adds hip BMD. | Per sex-specific SD higher medius/minimus HU: FOF OR 0.77 (CI 0.60–0.98); past-fall OR 0.82 (0.65–1.03), model 3. Area measures nonsignificant after adjustment. | Association models; discrimination, clinical-model increment and calibration NR. Past-fall association attenuated from model 2 OR 0.81 (0.66–0.98). | No prospective fall outcome or independent outcome validation. Participants were cognitively intact and independently ambulatory before injury. Functional muscle testing unavailable. |
| Wang 2020 (32) | 438 acute fractures / 316 controls before matching; matched on age, BMI and sex | Cross-sectional discrimination; gluteal attenuation, muscle area and CT-derived aBMD. | Gluteal HU AUC: neck fracture 0.88 (CI 0.85–0.92); intertrochanteric 0.95 (0.92–0.97). | All bone/muscle model: 0.912 / 0.958; model without muscle attenuation: 0.755 / 0.858. This is imaging-to-imaging comparison. | Apparent fracture-status discrimination. Future-risk calibration and external prognostic validation not established. |
| Li 2026 (33) | Fracture-control n=550 (283 fractures / 267 controls); any-fracture matched analysis n=320 (160/160). Index CT; gluteal fat at lower S2. | Age, sex, BMI matching; logistic models. Selected model 2: FN vBMD plus medius/minimus and combined maximus/medius/minimus IMAT. | Any-fracture AUC: combined-gluteal IMAT 0.896 (CI 0.862–0.930); FN vBMD 0.815 (0.770–0.861); selected model 2, 0.927 (0.899–0.954). | Imaging comparison; no full clinical-only comparator. Hosmer–Lemeshow P=0.83 for model 2. Calibration intercept/slope/plots NR; matched case-control fit is not population-risk calibration. | Apparent discrimination; resampling and independent outcome validation NR. Model 3 adds total-hip vBMD: AUC 0.927 (0.899–0.955), H–L P=0.85. Not post-index prognosis. |
| Pickhardt 2020 (35) | n=9,223; screening CT; median follow-up 8.8 years; 686 major / 219 hip fractures. Noncontrast 120-kVp CT. Prior fractures were not universally excluded. | L1 bone / L3 muscle attenuation; logistic ROC at 2, 5, 10 years; Cox models. CT-only, FRAX, CT+FRAX, and age/sex comparisons. | 2-year hip AUC: muscle 0.75 (CI 0.63–0.86); FRAX 0.73 (0.60–0.86); bone+muscle 0.76 (0.67–0.86); CT+FRAX 0.78 (0.68–0.88). | CT versus FRAX: P=0.73. Adding FRAX to CT: reported P=0.757; significant gain not shown. CT improved major-fracture discrimination over age/sex alone (2-year P=0.027). | Frozen extraction tools tested in a separate cohort; combined outcome models fitted here. Resampling correction and absolute-risk calibration NR; no independent post-index validation. |
| Liu 2024 (36) | Retrospective longitudinal case-control sample n=6,926, including 1,308 later hip fractures; baseline abdominal CT | Age-sex-matched control selection; univariable/multivariable CT models. Algorithm-training cohort was separate. | 10-year AUC: bone 0.702; muscle 0.603; subcutaneous fat 0.603; all three 0.733. CIs NR in performance tables. | Imaging-combination increase over bone alone is 0.031; no full clinical-only comparator. Calibration NR. | Outcome-model performance apparent; separate algorithm validation is not external prognostic-model validation. No post-index transportability test. |
| Wang 2022 (37) | Prospective second-fracture analysis; 45 events. Abstract reports 302 first-fracture participants; analyzed subsets vary with mobility/death exclusions. | Cox; cause-specific hazards; Fine–Gray sensitivity. Age, sex, diabetes and later Parker mobility used; additional aBMD adjustment. | Per SD lower gluteus medius/minimus HU: HR 1.68 (CI 1.20–2.35); after FN aBMD adjustment: 1.66 (1.18–2.30). | Adjusted association is not ΔAUC. Hosmer–Lemeshow testing applies to the separate cross-sectional logistic analysis, not calibrated prospective second-fracture risk. | No independent validation. Later mobility and post-baseline exclusions limit an index-time prediction claim; FN aBMD was null over the full horizon. |
| Wang 2024 (38) | Related CSHFE cohort; 45 events: 10 in year 1, 9 in year 2, 26 after year 2; median follow-up 4.5 years | Time-window Cox models; age, sex, BMI and diabetes in methods. Muscle-figure legend specifies pre-fracture Parker mobility. | FN aBMD year-1 HR 5.88 (CI 1.32–26.09); later gluteal attenuation associations. | ΔAUC, prospective risk calibration and external validation NR. Associations across windows do not establish differential drug or rehabilitation response. | Methods/figure covariate wording differs. Sparse early events and shared cohort preclude strong timing rules or independent replication. |

Abbreviations: aBMD, areal bone mineral density; AUC, area under the receiver operating characteristic curve; BMI, body mass index; CI, confidence interval; DXA, dual-energy X-ray absorptiometry; FN, femoral neck; HR, hazard ratio; HU, Hounsfield units; ICC, intraclass correlation coefficient; IMAT, intermuscular adipose tissue; OR, odds ratio; ROI, region of interest; SD, standard deviation; vBMD, volumetric bone mineral density. CCI, Charlson Comorbidity Index; CV, coefficient of variation; DBP, diastolic blood pressure; BUN, blood urea nitrogen; FOF, fear of falling; MFES, Modified Falls Efficacy Scale; PASE, Physical Activity Scale for the Elderly; PT, physical therapy; H–L, Hosmer–Lemeshow. NR is defined in Methods S1.4.

# Primary sources for Table S1

20. Eto S, Imanishi S, Komuta W, Oba Y, Hidaka N. Validation of Hounsfield units for preoperative bone mineral density assessment in intertrochanteric femoral fractures. J Orthop Sci. (2026) 31:444–449. doi: 10.1016/j.jos.2025.08.005

22. Fan J, Lv Y, Xu X, Zhou F, Zhang Z, Tian Y, et al. Evaluation of femoral head bone quality by Hounsfield units: a predictor of implant failure for intertrochanteric fractures after intramedullary nail fixation. Front Surg. (2023) 9:816742. doi: 10.3389/fsurg.2022.816742

23. Ye K, Hao Y, Dong Y, Hu Y, Qi J, Luo Y, et al. The predictive value of proximal femoral CT attenuation in intramedullary fixation failure of intertrochanteric fractures: a retrospective cohort analysis. Arch Osteoporos. (2026) 21:15. doi: 10.1007/s11657-025-01651-z

25. Bae SJ, Lee SH. Computed tomographic measurements of the psoas muscle as a predictor of mortality in hip fracture patients: muscle attenuation helps predict mortality in hip fracture patients. Injury. (2021) 52:1456–1461. doi: 10.1016/j.injury.2020.11.062

28. Yokoyama K, Ukai T, Ogawa M, Watanabe M. Preoperative computed tomography assessment of peri-hip muscles in patients with femoral neck fracture and its impact on postoperative walking function. BMC Musculoskelet Disord. (2025) 26:490. doi: 10.1186/s12891-025-08668-y

29. Achebe C, Cureton R, Rothberg D, Higgins T, Marchand L, Haller J. Preoperative quadriceps muscle quality predicts early ambulation after hip fracture surgery. J Orthop Trauma. (2026) 40:366–372. doi: 10.1097/BOT.0000000000003164

30. Ge Y, Li R, Tao X, Chen Y, Li T, Wang L, et al. Gluteal muscle density, but not size, is independently associated with fall risk in older adults with hip fractures: a cross-sectional study using opportunistic CT imaging. Maturitas. (2026) 211:108975. doi: 10.1016/j.maturitas.2026.108975

32. Wang L, Yin L, Zhao Y, Su Y, Sun W, Liu Y, et al. Muscle density discriminates hip fracture better than computed tomography X-ray absorptiometry hip areal bone mineral density. J Cachexia Sarcopenia Muscle. (2020) 11:1799–1812. doi: 10.1002/jcsm.12616

33. Li W, Zong R, Wu J, Wei M, Zhou W, Pu J, et al. Automatic phantom-less QCT predicts hip fracture risk: bone and muscle parameters comparison. Eur J Radiol. (2026) 195:112562. doi: 10.1016/j.ejrad.2025.112562

35. Pickhardt PJ, Graffy PM, Zea R, Lee SJ, Liu J, Sandfort V, et al. Automated abdominal CT imaging biomarkers for opportunistic prediction of future major osteoporotic fractures in asymptomatic adults. Radiology. (2020) 297:64–72. doi: 10.1148/radiol.2020200466

36. Liu D, Garrett JW, Perez AA, Zea R, Binkley NC, Summers RM, et al. Fully automated CT imaging biomarkers for opportunistic prediction of future hip fractures. Br J Radiol. (2024) 97:770–778. doi: 10.1093/bjr/tqae041

37. Wang L, Yin L, Yang M, Ge Y, Liu Y, Su Y, et al. Muscle density is an independent risk factor of second hip fracture: a prospective cohort study. J Cachexia Sarcopenia Muscle. (2022) 13:1927–1937. doi: 10.1002/jcsm.12996

38. Wang L, Yang M, Ge Y, Liu Y, Wang G, Su Y, et al. Risk prediction of second hip fracture by bone and muscle density of the hip varies with time after first hip fracture: a prospective cohort study. Bone Rep. (2024) 20:101732. doi: 10.1016/j.bonr.2023.101732

## Additional methodological sources

17. Keyak JH, Sigurdsson S, Karlsdottir GS, Oskarsdottir D, Sigmarsdottir A, Kornak J, et al. Effect of finite element model loading condition on fracture risk assessment in men and women: the AGES-Reykjavik study. Bone. (2013) 57:18–29. doi: 10.1016/j.bone.2013.07.028

39. Fine JP, Gray RJ. A proportional hazards model for the subdistribution of a competing risk. J Am Stat Assoc. (1999) 94:496–509. doi: 10.1080/01621459.1999.10474144

44. Moeller AR, Garrett JW, Summers RM, Pickhardt PJ. Adjusting for the effect of IV contrast on automated CT body composition measures during the portal venous phase. Abdom Radiol (NY). (2024) 49:2543–2551. doi: 10.1007/s00261-024-04376-8
